# Electrical Stimuli Are Anti-Apoptotic in Skeletal Muscle via Extracellular ATP. Alteration of This Signal in Mdx Mice Is a Likely Cause of Dystrophy

**DOI:** 10.1371/journal.pone.0075340

**Published:** 2013-11-25

**Authors:** Denisse Valladares, Gonzalo Almarza, Ariel Contreras, Mario Pavez, Sonja Buvinic, Enrique Jaimovich, Mariana Casas

**Affiliations:** 1 Centro de Estudios Moleculares de la Célula, Instituto de Ciencias Biomédicas, Facultad de Medicina, Universidad de Chile, Santiago, Chile; 2 Programa de Fisiología y Biofísica, Instituto de Ciencias Biomédicas, Facultad de Medicina, Universidad de Chile, Santiago, Chile; 3 Departamento de Ciencias Básicas y Comunitarias, Facultad de Odontología, Universidad de Chile, Santiago, Chile; University of Kansas School of Medicine, United States of America

## Abstract

ATP signaling has been shown to regulate gene expression in skeletal muscle and to be altered in models of muscular dystrophy. We have previously shown that in normal muscle fibers, ATP released through Pannexin1 (Panx1) channels after electrical stimulation plays a role in activating some signaling pathways related to gene expression. We searched for a possible role of ATP signaling in the dystrophy phenotype. We used muscle fibers from *flexor digitorum brevis* isolated from normal and mdx mice. We demonstrated that low frequency electrical stimulation has an anti-apoptotic effect in normal muscle fibers repressing the expression of Bax, Bim and PUMA. Addition of exogenous ATP to the medium has a similar effect. In dystrophic fibers, the basal levels of extracellular ATP were higher compared to normal fibers, but unlike control fibers, they do not present any ATP release after low frequency electrical stimulation, suggesting an uncoupling between electrical stimulation and ATP release in this condition. Elevated levels of Panx1 and decreased levels of Cav1.1 (dihydropyridine receptors) were found in triads fractions prepared from mdx muscles. Moreover, decreased immunoprecipitation of Cav1.1 and Panx1, suggest uncoupling of the signaling machinery. Importantly, in dystrophic fibers, exogenous ATP was pro-apoptotic, inducing the transcription of Bax, Bim and PUMA and increasing the levels of activated Bax and cytosolic cytochrome c. These evidence points to an involvement of the ATP pathway in the activation of mechanisms related with cell death in muscular dystrophy, opening new perspectives towards possible targets for pharmacological therapies.

## Introduction

ATP was considered for a long time as a molecule exclusively involved in cell energy and metabolism. Nevertheless, in the past years, ATP has been shown to be an important extracellular messenger for autocrine and paracrine signaling [1].

There are two families of receptors for extracellular nucleotides: P2X and P2Y. P2X receptors are ion channels activated by ATP which induce fast and non-selective inward currents of Na^+^ and Ca^2+^ [2]. P2Y receptors are GPCR activated by ATP, ADP, UTP or UDP [3]. To date, seven mammalian P2X subtypes (P2X1-7) and eight mammalian P2Y subtypes (P2Y1,2,4,6,11,12,13,14) have been cloned and pharmacologically characterized [2]–[4].

ATP has been described as a regulator of inflammation, in embryonic and stem cell development, in ischemia and in several other processes [5]–[6]. In skeletal muscle, ATP has been implicated in the regulation of proliferation, differentiation and regeneration [7]–[8] and also in promoting the stabilization of the neuromuscular junction [9]. We have described that ATP is released trough Pannexin-1 (Panx1) channels by muscle cells after electrical stimulation and plays a crucial role in the activation of signaling pathways that lead to transcription of several genes [10]–[11]. Moreover, in adult muscle fibers, this signaling pathway mediates some of the muscle effects of nerve activity related with the process of muscle cell plasticity [11]–[12].

A number of skeletal muscle pathologies have been associated with alterations in the metabolism of extracellular ATP, with changes in the sensitivity towards ATP and with altered expression of purinergic receptors. One of them is the Duchene Muscular Dystrophy (DMD) [13]–[14], that is caused by the absence of functional dystrophin [15], a cytoskeleton protein mainly expressed near the cytosolic face of the plasma membrane [16]. In normal skeletal muscle, dystrophin is associated with a complex of glycoproteins known as dystrophin-associated proteins (DAPs), providing a linkage between the extracellular matrix and cytoskeleton [17]. Lack of dystrophin in dystrophic muscle results in loss of the complex integrity and allegedly impairs the stability of the plasma membrane causing mechanical stress fragility and an increase in Ca^2+^ permeability [18]. Nevertheless, the pathophysiology of this muscular dystrophy cannot be explained by this increased mechanical fragility alone and a role for dystrophin and DAPs has been suggested as part of a protein signaling complex involved in cell survival [19].

Several evidences relate ATP signaling with the abnormal Ca^2+^ homeostasis observed in dystrophic muscle, suggesting an important role in the pathogenesis of this disease [13]–[14]. In myoblasts of a dystrophin-negative muscle cell line, exposure to extracellular ATP elicited a strong increase in cytoplasmic Ca^2+^ concentrations, compared to control myoblasts. This increased susceptibility to ATP was due to changes in expression and function of P2X receptors and proposed to be a significant contributor to pathogenic Ca^2+^ entry in dystrophic mouse muscle [13]. Also, the stimulation of P2 receptors with ATP continuously released in response to stretching has been proposed to constitutively activate the plasma membrane Na^+^/H^+^ exchanger (NHE), contributing to the sustained increase in intracellular Ca^2+^ [14].

Necrosis is probably a major contributor to muscle fiber loss in DMD and there is extensive experimental support for necrotic cell death in dystrophin-deficient muscles [20]–[23]. Nevertheless, several studies suggest that during the phase of acute muscle degeneration in the mdx mouse, apoptosis precedes necrosis [24]. Early evidences of apoptotic muscle-fiber loss in muscular dystrophy have been provided in mdx mice, a murine model of DMD [25]–[28]. Later on, nuclei that seem to die by apoptosis were found in dystrophin-deficient muscles in mdx mice and human DMD patients [24], [29].

Apoptosis plays important roles in both developing and adult human skeletal muscle and has been proposed to partially remove damaged cells after eccentric exercise [30]. Additionally, elevated apoptotic signaling has been reported in skeletal muscle during aging, stress-induced states and disease; relating it to muscle dysfunction, degradation, and atrophy [31]. A large number of internal and external signals regulate the expression of genes that control the initiation of apoptosis [32]. These genes are related to the expression of proteins of the Bcl-2 family, which can have either pro- or anti-apoptotic activities and regulate the intrinsic pathway of apoptosis by controlling the permeabilization of the outer mitochondrial membrane [33].

In the present work, we show that basal extracellular ATP levels are increased in *flexor digitorum brevis* (*fdb)* muscle fibers from mdx mice. This increase appears to be due to enhanced ATP release via Panx1 channels in the mdx fiber. Extracellular ATP induces altered gene expression in mdx fibers, increasing expression of genes related with apoptosis. These results suggest that the anti-apoptotic role of electrical stimulation is lost in dystrophic fibers and that the enhanced ATP signaling observed in mdx fibers could be related with muscle fiber death.

## Materials and Methods

### Adult Fiber Isolation

5–7 wk old mice were used throughout this work. All protocols were approved by the Bioethics Committee, Faculty of Medicine, Universidad de Chile. Mdx mice were used as a model of DMD and C57Bl/6 as normal controls. Procedure of isolation was described previously [12]. Briefly, isolated muscle fibers from mouse flexor digitorum brevis (FDB) were obtained by enzymatic digestion of the whole muscle with collagenase type 2 (Worthington, Lakewood, NJ) for 90 min at 37°C. Afterwards, the muscle was mechanically dissociated with fire polished Pasteur pipettes. Isolated fibers were seeded in matrigel coated cover slips in Dulbecco’s modified Eagle medium (Invitrogen) supplemented with 10% horse serum (Invitrogen). The fibers were used for experimentation no more than 20 hours after isolation.

### Muscle Fiber Stimulation

Electrical stimulation (ES) of muscle fibers was performed as previously reported [12]. In brief, cells were washed with Krebs buffer (145 mM NaCl, 5 mM KCl, 1 mM CaCl_2_, 1 mM MgCl_2_, 5.6 mM glucose, 10 mM HEPES, pH 7.4) and let to rest for 60 min prior stimulation. Muscle fibers in a dish were stimulated with 270 pulses of 0.3 ms duration at 20 Hz with a stimulation device that consists of a row of six platinum wires intercalated 0.5 cm apart with alternate polarity across a circular plastic holder that fits in the dish. The pulses were delivered by a GRASS S48 stimulator (Axon instruments). Mechanical stimulation (MS) was evoked by changing the Dulbecco’s modified Eagle medium for a Krebs solution as described previously [34]. For ATP stimulation, 10 to 100 µM exogenous ATP (Sigma-Aldrich) was added directly to the fibers in the culture media. Isolated fibers were incubated with specific agonist for P2Y1R (MRS2365, 100 nM, Tocris) or P2Y2R (UTP-γS, 10 µM, Tocris) and the samples for RNA extraction were recovered at the times indicated. Additionally, the fibers were incubated with inhibitors for hemichannels (oleamide 100 mM, Sigma-Aldrich) or Pannexin (carbenoxolone 5 µM, ^10^Pnx1 100 µM) for 30 minutes before the stimulation.

### ATP Detection by Luciferin/Luciferase Assay

50 µl of extracellular samples were added to 20 µl of CellTiter-Glo® Luminescent Cell Viability Assay (Promega). After 10 minutes incubation at dark, samples were quantified in a luminomiter. In parallel, a standard curve from 1 fmol to 100 pmol ATP was performed using the same kit. Lineal range was obtained from 100 fmol to 10 pmol ATP. Samples lectures were interpolated in standard curve to detect ATP concentrations in each condition. Data were presented as pmol extracellular ATP/µg total RNA and the ratios between experimental versus control points were reported. Normalization by total RNA instead of total protein was chosen because adult muscle fibers were seeded on a matrigel coated surface (containing a large amount of protein), which may affect the protein determination associated to fibers only.

### Calcium Signals Measurement

Isolated FDB fibers were incubated 30 min with 5 µM Fluo3-AM at room temperature in standard Krebs buffer. Electrical stimulation (270 pulses of 0.3 ms duration at 20 Hz) was applied with a couple of platinum electrodes connected through an isolation unit to a stimulator. Images series during stimulation experiments were obtained with a confocal microscope (Carl Zeiss Axiovert 135 M, LSM Microsystems). After excitation with a 488-nm wavelength Argon laser, the fluorescence images were collected every 1.0–2.0 s (corresponding to exposure time for each image) and analyzed frame by frame. The average cell fluorescence, F, was calculated for each image on an outline of the cell and normalized to its initial or pre-intervention value F_0_ as (F–F_0_)/F_0_.

### Real Time PCR

Total RNA was obtained from skeletal muscle fibers employing Trizol reagent (Invitrogen, Corp.) according to manufacturer’s protocol. cDNA was prepared with SuperScript (Invitrogen), according to manufacturer’s protocol. Quantitative PCR was performed in Mx3000P® Thermocycler (Stratagene, La Jolla, CA, USA) using the Brilliant III Ultra-Fast QPCR & QRT-PCR Master Mix amplification kit (Agilent Technologies, Santa Clara, CA, USA). Forward and reverse primers were:

TNIS 5′- GAGGTTGTGGGCTTGCTGTATGA-3′ and ’-GGAGCGCATATTAGGGATGT-3′

TNIF 5′- AGGTGAAGGTGCAGAAGAGC -3′ and 5′- TTGCCCCTCAGGTCAAATAG -3′

bax 5′-GCTGACATGTTTGCTGATGG-3′ and 5′-GATCAGCTCGGGCACTTTAG-3′

bim 5′-CGACAGTCTCAGGAGGAACC-3′ and 5′-CATTTGCAAACACCCTCCTT-3′

PUMA 5′- GCCCAGCAGCACTTAGAGTC -3′ and 5′- GGTGTCGATGCTGCTCTTCT -3′

bcl2 5′- AGTACCTGAACCGGCATCTG -3′ and 5′- GCTGAGCAGGGTCTTCAGAG -3′

18S rRNA 5′-GGGCCCGAAGCGTTTACTTT-3′ and 5′-TTGCGCCGGTCCAAGAATTT-3′.

Expression values were normalized to 18S and were reported in units of 2^−ΔΔCT^ ± S.E as described [35]. CT value was determined by MXPro software when fluorescence was 25% higher than background. PCR products were verified by melting-curve analysis.

### Isolation of Skeletal Triads or T-tubules

Preparation of triad-enriched fractions from back and limbs muscles derived from 6–8 week old BalbC mice were performed as previously standardized in our laboratory for frog and rabbit muscles, using differential centrifugation [36]–[38].

### Co-immunoprecipitation Assay and Immunoblot

Triad-enriched fractions were solubilized for 1 h in 200 µl of lysis buffer (20 mM Tris-HCl pH 7.4, 0.1% Nonidet P-40, 5 mM EDTA pH 8, 10 mM EGTA pH 7.8, 140 mM NaCl, 10% glycerol and protease inhibitors). A 20 min-15000 g supernatant fraction was incubated 30 min with 10 µg A/G agarose as a pre-clearing strategy. The beads were spun down by centrifugation and washed 3 times with 200 µl of washing buffer (25 mM HEPES pH 7.5, 0.2% Nonidet P-40, 140 mM NaCl, 0.1% BSA, 10% glycerol and protease inhibitors). After the pre-clearing step, the whole cell extracts were incubated for 4 hours with the correspondent antibody and then incubated 30 min with 50 µg A/G agarose beads. The beads pellet was washed 3 times with washing buffer. Proteins were resolved by SDS-PAGE in 7–10% gels, transferred to polyvinylidene difluoride filters and blotted with the corresponding antibody.

### Western Blot Analysis

Stimulated cells were lysed in 60 µl of ice-cold lysis buffer containing 20 mM Tris-HCl, pH 7.4, 1% Triton X-100, 2 mM EDTA, 10 mM Na_3_VO_4_, 20 mM NaF, 10 mM sodium pyrophosphate, 150 mM NaCl, 1 mM PMSF and a protease inhibitor mixture (Roche Applied Science). The cell lysates were sonicated for 1 min, incubated on ice for 20 min, and centrifuged to remove debris. Protein concentration of the supernatants was determined with Coomassie Plus™ Protein Assay (Termo Scientific) using bovine serum albumin as standard. The lysate proteins were suspended in denaturizing buffer (62,6 mM Tris-HCl (pH 6.8); 2% Sodium Dodecyl Sulfate (SDS); 0.01% Bromophenol blue; 10% glicerol and 100 mM DTT), separated in 10% SDS-polyacrylamide gels, and transferred to polyvinylidene difluoride membranes. The membranes were blocked at room temperature for 1 h in Tris-buffered saline containing 3% fat-free milk, with 0.5% Tween 20, and then incubated overnight with the primary antibody for Dystrophin (1∶1000, Novocastra Laboratories Ltd), α1s-subunit of Cav1.1 (1∶4000, Affinity Bioreagents), Pannexin-1 (1∶2000, donation from Dr. Juan Carlos Sáez laboratory, Universidad Católica de Chile), P2Y1 (1∶2000, donation from Dr. Alfonso González laboratory, Universidad Católica de Chile) and P2Y2 (1∶2000, Zymed Laboratories). After washing with Tris-buffered saline, the membranes were incubated with the secondary HRP-conjugated anti-rabbit and anti-mouse antibodies (Pierce Biotechnology) at room temperature for 1.5 hours. The immunoreactive proteins were detected using ECL reagents (Amersham Biosciences) according to the manufacturer’s instructions.

### Immunofluorescence

Fibers plated on 35 mm coverslips were stimulated with 100 µM of ATP for 6 hours. Fiber were washed with PBS and then fixed with paraformaldehyde (Electron Microscopy Science, Hatfield, PA, USA) at 4% in PBS for 10 min at room temperature. Then, fibers were rinsed with PBS and permeabilized with Triton X-100 0.1% in PBS, rinsed with PBS and blocked with PBS-1% BSA for 1 h at room temperature. Monocolonal antibodies against activated Bax NT (1∶100, Upstate) and cytochrome c (1∶100, Cell Signaling) were incubated at 4°C overnight. Fibers were washed and then incubated for 1 hour with Alexa Fluor-488 anti-mouse antibody (Molecular Probes Invitrogen). The samples were mounted in Dako anti fading reactive (Dako, Denmark) and stored at 4°C until use.

### Image Acquisition and Processing

All images were acquired with a Carl Zeiss Axiovert 135 M, LSM Microsystems, Apo Plan 63X, NA 1.4. Images deconvolution and processing were performed using Image J software (NIH).

### Statistical Analysis

Data of *n* experiments were expressed as mean ± S.E.M. The significance of difference among treatments was evaluated using a *t* test for unpaired data or analysis of variance followed by Dunnett’s post test for multiple comparisons or by one-way ANOVA test followed by Tukeýs post test. A *p* value<of 0.05 was considered statistically significant.

## Results

### ATP Release through Pannexin-1 Channels is Increased in mdx Muscle Fibers

**Figure 1:**
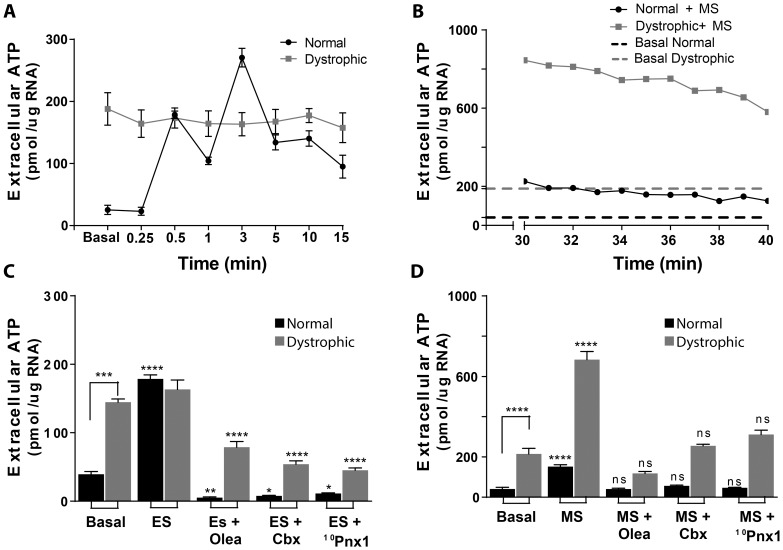
ATP release differs in normal versus dystrophic muscle fibers. (A) Isolated muscle fibers from C57Bl/6 and mdx mice were electrically stimulated (ES) with 270 pulses, 0.3 ms each at 20 Hz and ATP release was measured at the times indicated. Dystrophic fibers presented higher levels of basal extracellular ATP (before stimulation) than normal fibers and did not show the typical two peaks of ATP release after ES observed in normal fibers (n = 6). (B) Isolated fibers were subjected to change of culture media for Krebs solution (MS) and ATP release was measured 30 minutes after the stimulus. Dotted lines indicate the basal levels of ATP release, set 4 hours after MS. MS induced ATP release in both normal and dystrophic fibers, reaching higher levels (both, total and as fold of change versus its control) in dystrophic fibers. (C) Quantification of maximum ATP release (first peak) induced by ES in the presence of different Pannexin inhibitors. 100 µM oleamide (Olea), 5 µM carbenoxolone (Cbx) and 100 µM ^10^Pnx1 prevented ES-induced ATP release in normal fibers and decreased basal levels of ATP release in dystrophic fibers. (D) Same inhibition was observed in MS-induced ATP release in both fiber types with these inhibitors. ATP release values presented correspond to means of ATP release values during the 10 min of measurement shown in (B). Data were expressed as mean ± S.E. from n = 4–6 for the different inhibitors. *p<0.05,***p<0.001,****p<0.001 compared with basal level for each fiber type or with the experimental point without inhibitor.

It has been previously reported that mechanical stimulation induces ATP release in several cell types [34], and our group has shown that in adult muscle fibers, electrical stimulation induces ATP release trough Pannexin-1 (Panx1) channels [11]. In order to study the differences related to ATP signaling between normal and dystrophic fibers, we measured the levels of extracellular ATP in isolated fibers from flexor *digitorum brevis muscles (fdb*) from C57/Bl6 and mdx mice under these two types of stimulation. We measured ATP release in muscle fibers after electrical stimulation with 270 pulses (0.3 ms each) at 20 Hz and we observed that mdx muscle fibers showed increased initial (pre-stimulation) values of ATP release (145±5 pmol/µg RNA in dystrophic vs 40±4 pmol/µg RNA in normal fibers, p<0.0001) but did not show the typical two peaks of ATP release observed at 30 s and 3 min after the stimulus in control muscle fibers (Fig. 1A). We next studied ATP release 30 min after replacement of culture media for Krebs buffer. This manipulation constitutes a mechanical stimulus that itself induced ATP release (Fig. 1B). We observed that mechanical stimulation increased ATP release in both control and dystrophic muscle fibers but reached much higher values in dystrophic muscle cells, increasing 2.5±0,3 times and 3.5±0,1 times vs basal conditions, in normal vs dystrophic fibers respectively (p<0.001) (Fig. 1B and D). Basal ATP release in this experiment was established for both fiber genotypes by measuring ATP release from one and up to 4 h after medium replacement (first bars in Fig. 1 D and dotted lines in Fig. 1B), time at which release remained stable (not shown). Basal ATP release in dystrophic fibers was 215±28 pmol/µg RNA, a much higher value compared with 41±7 pmol/µg RNA for normal fibers.

Several studies have described that ATP is released to the extracellular media through connexin or pannexin channels [39]–[42]. Moreover, there are evidences implicating Panx1 in ATP release after electrical stimulation in primary cultures of skeletal myotubes [10] and in adult muscle fibers [11]. To elucidate the participation of Panx1 as the route for ATP release in the dystrophic muscle context, we used three different pharmacological inhibitors: 100 µM oleamide, a non-selective blocker of hemichannels; 5 µM carbenoxolone, a blocker of Panx1 channels at this concentration [40] and 100 µM ^10^Panx1, an inhibitor peptide, specific for Panx1 [43]. Treatment of fibers with each of these inhibitors decreased the levels of ATP release in both, C57Bl/6 and mdx muscle fibers after electrical (Fig. 1C) and mechanical stimulation (Fig. 1D). These results indicate that Panx1 is the principal route of ATP release in control and mdx muscle fibers.

### ATP-dependent Excitation-transcription Coupling is Altered in Adult Dystrophic Muscle Fibers

**Figure 2:**
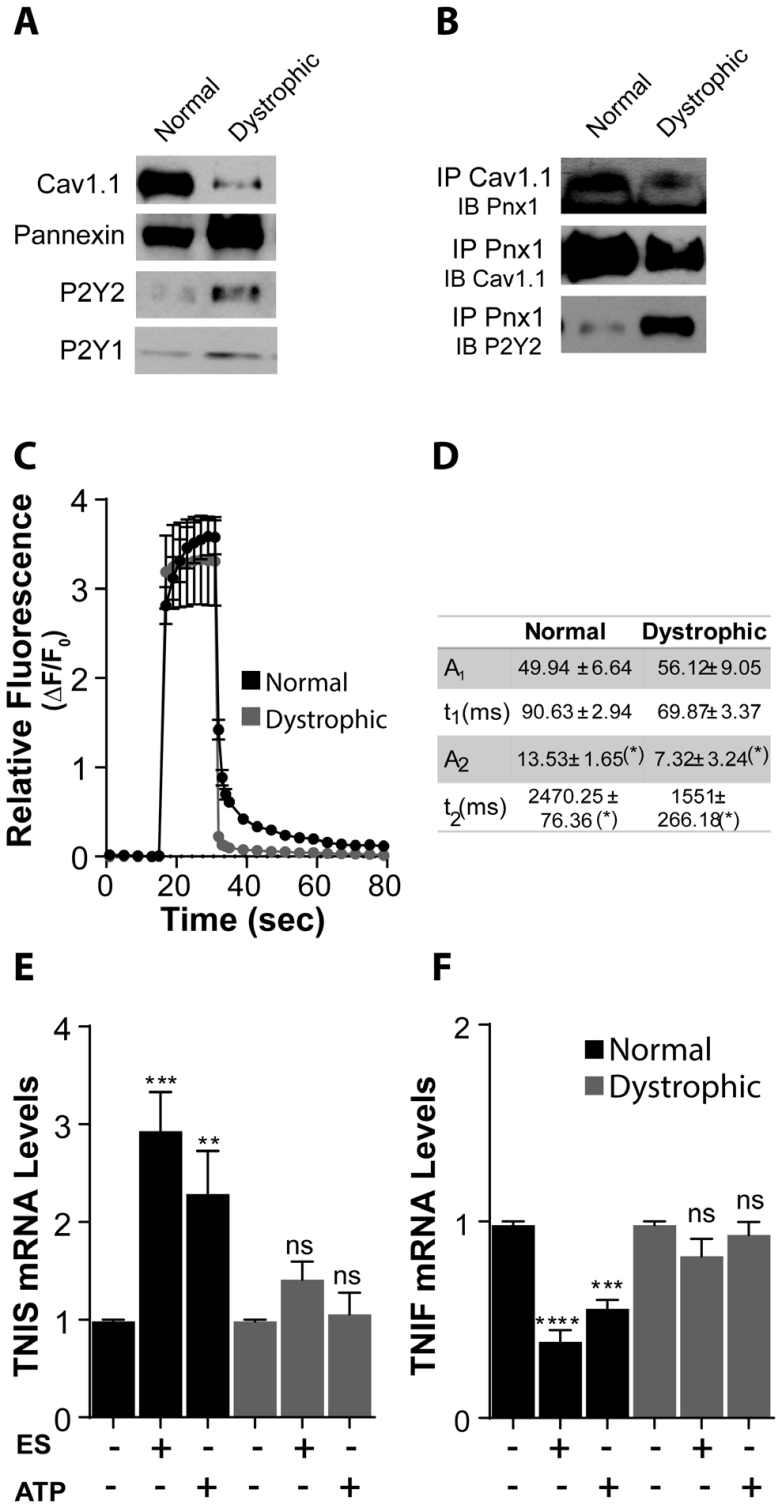
Excitation-Transcription (E-T) coupling is altered in dystrophic muscle fibers. (A) In triad fractions obtained from normal and dystrophic muscles we observed a decrease in the expression of Cav1.1 (antibody against α1 s subunit) and an increase in the expression of Panx1, P2Y2 and P2Y1 in dystrophic compared to normal (n = 4). (B) In these fractions, we found that Cav1.1 co-immunoprecipitated with Panx1 but in a lesser extent in the dystrophic muscle fractions. Panx1 also co-immunoprecipitated with P2Y2 in both muscle types but the signal appears to be much weaker in normal compared to dystrophic muscles (n = 3). (C) Isolated fibers from normal and dystrophic mouse were loaded with Fluo3-AM and then stimulated with 270 pulses (0.3 ms each) at 20 Hz. We observed in both fiber types a first fast calcium signal related to contraction when pulses were applied followed by a slower signal, observed as a delayed return to basal fluorescence levels after stimulation (n = 6). (D) Values of amplitude (A2) and time constant (τ2) for the double exponential fit of the post-tetanic calcium signal presented in (C) were significantly lower for mdx fibers compared to normal ones. (E,F) mRNA levels of the slow isoform of Troponin I (TNIS) were increased in normal fibers 4 h after 20 Hz electrical stimulation or addition of 100 µM ATP (E), whereas those of the fast isoform of Troponin I (TNIF) were decreased by these two types of stimulus (F). Neither 20 Hz stimulation nor ATP addition induced any change in mRNA levels of Troponin I isoforms in dystrophic muscle fibers (n = 3 for each gene). Data are expressed as mean ± SE. *p<0.05,***p<0.001,****p<0.0001.

It has been shown that Panx1 is present in both sarcolemma and T-tubules of skeletal muscle fibers [11], where it can interact with the L-type Ca^2+^ channel Cav1.1. This interaction is important for activation of ATP release after electrical stimulation in control fibers, because blocking Cav1.1 prevents ATP release after electrical stimulation [11]. We studied the protein levels of Cav1.1, Panx1, P2Y1 receptor and P2Y2 receptor by western blot in subcellular fractions enriched in triads obtained from control and dystrophic muscles from 5 week old mice. It can be observed that lower levels of Cav1.1, and higher levels of Panx1, P2Y1 and P2Y2 were found in samples from dystrophic mice (Fig. 2A) compared to control. These increased values of Panx1 channels could explain the increased ATP release values observed in basal conditions in mdx fibres.

To study the interaction between Panx1, Cav1.1 and P2Y2, we performed co-immunoprecipitation assays with protein extracts from triad fractions as those of figure 2A. We observed that interaction between Panx1 and Cav1.1 was weaker in dystrophic muscles compared to control, whereas interaction between Panx1 and P2Y2 appears to be stronger.

Post-tetanic IP_3_-dependent Ca^2+^ signals are induced in adult muscle cells after electrical stimulation at 20 Hz. These Ca^2+^ signals participate in activation of genes related to muscle plasticity, increasing mRNA levels of slow isoform of Troponin I (TnIs) and decreasing those of the fast isoform of this gene (TnIf) [12]. As above mentioned, ATP release plays a crucial role in activation of this IP_3_-dependent signal and in the associated transcriptional changes that occur after electrical stimulation in both primary myotubes [10] and in adult muscle fibers [11].

As ATP release after electrical stimulation was impaired in mdx muscle fibers, we measured the Ca^2+^ signals evoked by electrical stimulation in mdx and control muscle fibers. We can observe in Fig. 2C that there was a significant reduction of the post-tetanic component of Ca^2+^ signal, as can be seen by the faster return to basal fluorescence levels in traces corresponding to mdx fibers compared to those of control fibers. We have previously shown that the post-tetanic Ca^2+^ signal can be fitted by a two exponential decay, with the slower component (characterized by the values of A2 and τ2) corresponding to that dependent on IP_3_R [12]. In Fig. 2D we can observe that both, the value of amplitude A2 and that of the time constant τ2 were significantly lower for mdx fibers compared to control, indicating that IP_3_-dependent Ca^2+^ signals were smaller in dystrophic muscle cells. Accordingly, transcriptional changes observed after 20 Hz electrical stimulation in control muscle fibers on Troponin I (TnI) genes were not observed in dystrophic ones (Fig. 2 E and F). As IP_3_-dependent Ca^2+^ signals and the transcriptional changes in TnI genes observed after 20 Hz electrical stimulation were dependent on ATP release, these results were in agreement with the data presented in Fig. 1 showing that no additional ATP release takes place in mdx muscle fibers after electrical stimulation.

We have shown before that addition of exogenous ATP (in the absence of electrical stimulation) induces the same transcriptional changes in TnI gene’s isoforms that 20 Hz electrical stimulation does [11]. For this reason we searched to know if stimulation of mdx muscle fibers with external ATP could induce transcriptional changes in TnI isoforms. We observed that addition of 100 µM of ATP induced an increase in mRNA levels of TnIs and a decrease in those of TnIf in control fibers, without inducing any change in mdx fibers, showing that, besides presenting no electrically stimulated ATP release, mdx fibers shows defaults downstream ATP stimulation. These data suggest that the impairment of Cav1.1-Panx1 functional interaction in dystrophic muscles reduces the IP_3_ dependant component of Ca^2+^ signals and the gene expression related to fast-to-slow transition.

### Electrical Stimulus has an Anti-apoptotic Effect in Normal Muscle Fibers

**Figure 3:**
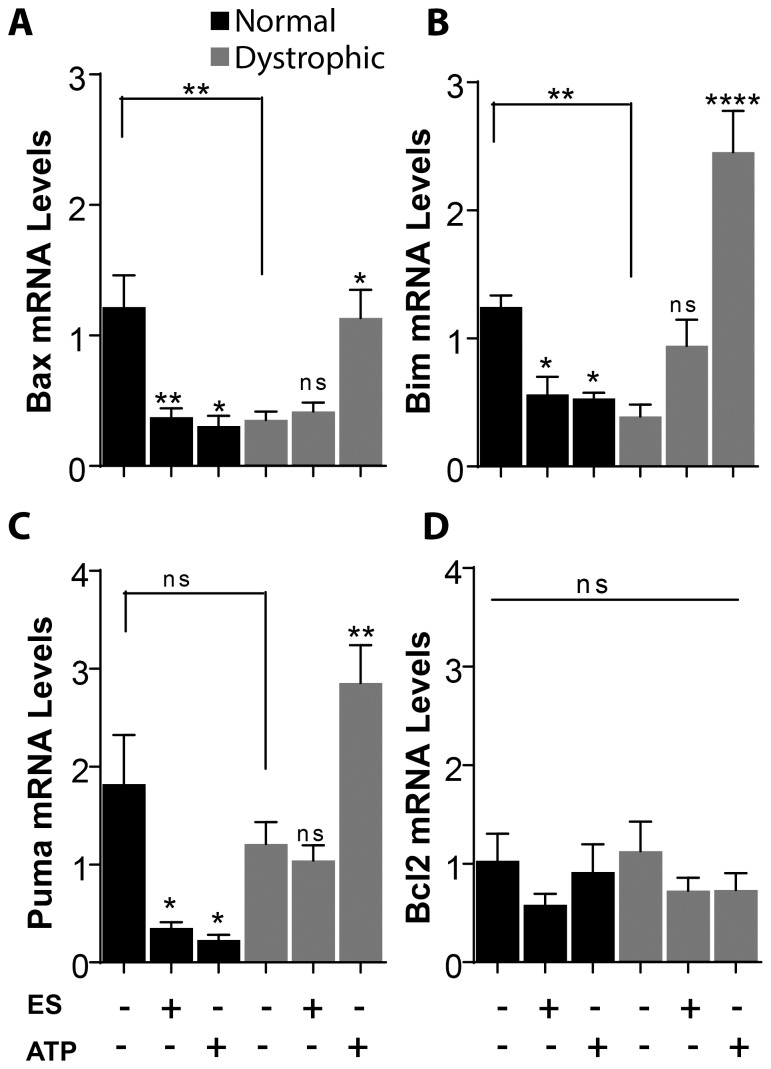
ATP has an anti-apoptotic effect in normal fibers but a pro-apoptotic effect in dystrophic fibers. Isolated fibers were electrically stimulated at 20 µM external ATP. In normal fibers, mRNA levels of the pro-apoptotic genes Bax (A), Bim (B) and PUMA (C) were reduced 1 h after 20 Hz and ATP stimulation. 20 Hz stimulation induced no changes in mRNA levels of these genes in dystrophic fibers while ATP induced an increase (A, B and C). (D) mRNA levels of the anti-apoptotic gene Bcl2 did not change with the stimuli used in neither normal nor dystrophic fibers. Basal mRNA levels of Bax and Bim were reduced in dystrophic fibers (A and B), while no change in basal mRNA levels of PUMA and Bcl2 were observed (C and D) (n = 6–9 for each gene). Data were expressed as mean ± SE. *p<0.05,**p<0.01,****p<0.0001.

Many genes of the Bcl2 family are related with the initiation of apoptosis. Among them, Bim has been described as an activator of apoptosis due to its capability of directly activating Bax, a protein that induces mitochondrial outer membrane permeabilization [33], [44]–[45]. We studied the expression of these different genes related with apoptosis after electrical stimulation at 20 Hz and we observed a significant decrease in Bax, Bim and Puma mRNA in control fibers (Fig. 3A, B and C). mRNA levels for the anti-apoptotic Bcl2 protein did not show statistically significant differences after electrical stimulation (Fig. 3D). Electrical stimulation in dystrophic fibers, on the other hand, induced no significant changes in mRNA levels of any of the genes studied (Fig. 3). It must be pointed that mdx fibers showed lower basal levels of Bax (0.4±0.1 times compared to normal fibers, p<0.01) and Bim (0.4±0.1 times, p<0.01), not reaching statistically significance for Puma. This result is in agreement with the elevated basal levels of extracellular ATP observed in mdx muscle fibers (Fig. 1), suggesting that the signaling pathway leading to the decrease in apoptotic genes in control fibers could be already activated in dystrophic fibers by their increased levels of basal ATP release.

### ATP Signaling is Related with Increased Expression of Cell Death Genes in mdx Fibers

To assess the participation of ATP in the induction of cell death in normal and dystrophic fibers we studied the expression of the different genes related with apoptosis after addition of 100 µM of external ATP. First, we stimulated the normal and dystrophic fibers with 100 µM of ATP to characterize the time response and then tested different ATP concentrations while measuring Bax and Bim mRNA levels (showed for Bax mRNA levels in Fig. S1). We study the fibers one hour after ATP stimulation because we found that at this time, Bax expression reach the maximal level in dystrophic fibers (Fig. S1). We also found that the minimum concentration needed to induce changes in the expression of apoptotic genes in control fibers was 100 µM, so, even if in dystrophic fibers 50 µM was enough to induce transcriptional changes in the genes studied, we decide to continue with 100 µM in order to be able to compare the effect of ATP stimulation between normal and dystrophic fibers. We observed that stimulation with 100 µM external ATP produced a decrease in mRNA levels of Bax, Bim and Puma in control muscle fibers (Fig. 3A, B and C), similarly to what was seen with 20 Hz electrical stimuli; whereas an increase in mRNA levels of Bcl2 was observed in this case (Fig. 3D). On the contrary, when dystrophic muscle fibers were stimulated with external ATP, we observed the opposite effect: an increase in mRNA levels of Bax, Bim and Puma (Fig. 3 A, B and C), without significant changes in Bcl2 mRNA levels (Fig. 3D).

Taken together, these results suggest that ATP and electrical stimulation in normal fibers have a protective effect against cell death. Nevertheless, in dystrophic fibers, ATP stimulation induces an increase in the expression of pro-apoptotic genes and, by activating an apoptotic pathway, could be participating in the loss of fibers in DMD. Even if we show before that mdx fibers presented a lower basal mRNA levels for Bax and Bim, the fact of adding more ATP probably makes the fibers to reach an ATP threshold at which the anti-apoptotic effect is lost and an apoptotic pathway became activated instead.

### mdx Fibers Treated with External ATP Show Apoptotic Signs

As Bax is the protein responsible for initiation of cell death, we searched to know if it became activated in dystrophic and control muscle fibers after stimulation with external ATP. We evaluated activated Bax after 6 hours of ATP stimulation. This is a longer time of ATP stimulation than those used to evaluate transcriptional changes of Bax (Fig. 3), because changes in protein were not seen when using only one hour of stimulation. In order to avoid ATP degradation by cellular ecto-nucleotidases, we renew ATP addition to the fibers every hour during the first 3 hours of incubation (i.e. 3 times).

**Figure 4:**
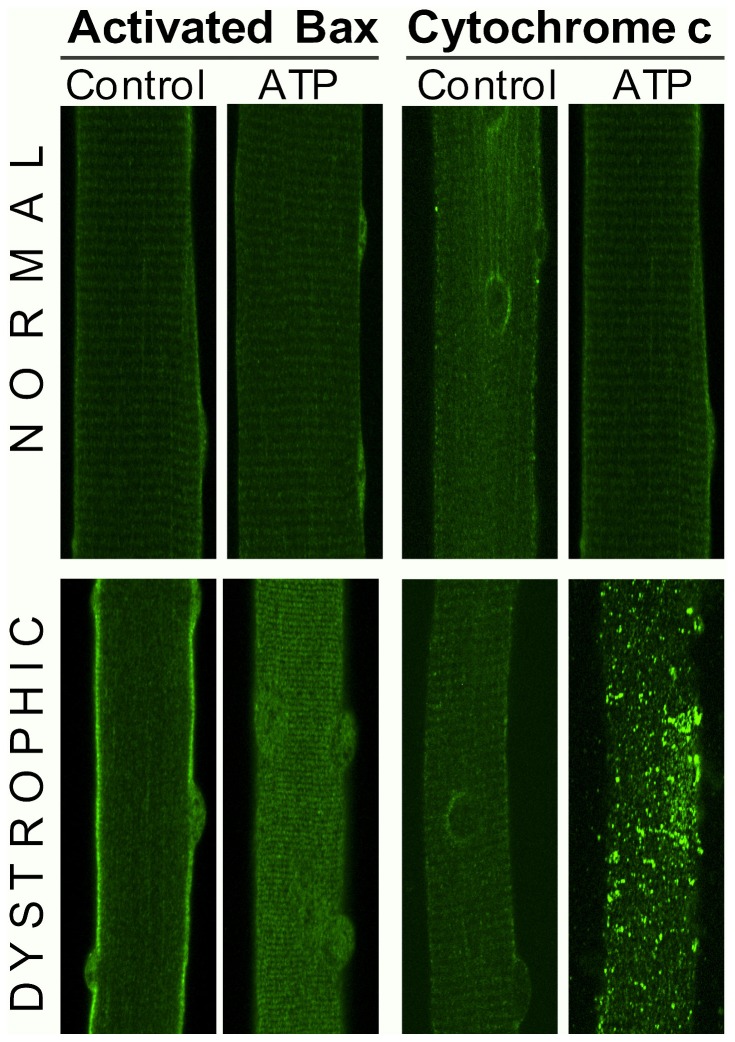
ATP stimulation induced activation of Bax and cytochrome c release. Images from 1 µm confocal slices of immunostaining for activated Bax (left panels) and citochrome c (right panels) in normal (upper) and dystrophic fibers (lower) were performed in adult muscle fibers treated with 100 µM ATP for 6 h. We observed that ATP stimulation did not alter the basal levels of activated Bax nor induced cytochrome c release in normal fibers. On the other hand, in dystrophic fibers, ATP stimulation induced an increase in the levels of activated Bax and the release of cytochrome c into the cytosol. For each condition a representative image from three different experiments is presented (n = 4).

In the immunofluorescene images using an antibody that recognizes the activated form of Bax [46], we found that ATP stimulation induced no changes neither in the level of this protein nor in its localization (Fig. 4 upper panels at left), showing a striated localization pattern.

Differing form normal fibers, in dystrophic fibers activated Bax appears to be mainly at the sarcolemma in basal conditions. After ATP stimulation, the amount of activated Bax increased and its intracellular localization changes to a more striated pattern suggesting mitochondria localization (Fig. 4 lower panels at left).

Cytochrome c, that normally localize into the mitochondria, can be released to the cytosol in response to a pro-apoptotic signal, where it can in turn activate or amplificate the apoptosis pathway [47]. Cytochrome c showed a striated and perinuclear pattern, according to mitochondria localization, in control and dystrophic fibers in basal conditions (Fig. 4 right panels). After ATP stimulation, in control fibers we observed no changes in intensity or in localization of this protein (Fig. 4, right upper panels). However, in dystrophic fibers, ATP stimulation seems to induce an increase in levels of cytochrome c, that localize now in the cytosol forming large aggregates (Fig. 4, right lower panels), according with activation of cell death.

### Activation of P2Y Receptors Induces an Increase in Expression of Cell Death Genes in mdx Muscle Fibers

**Figure 5:**
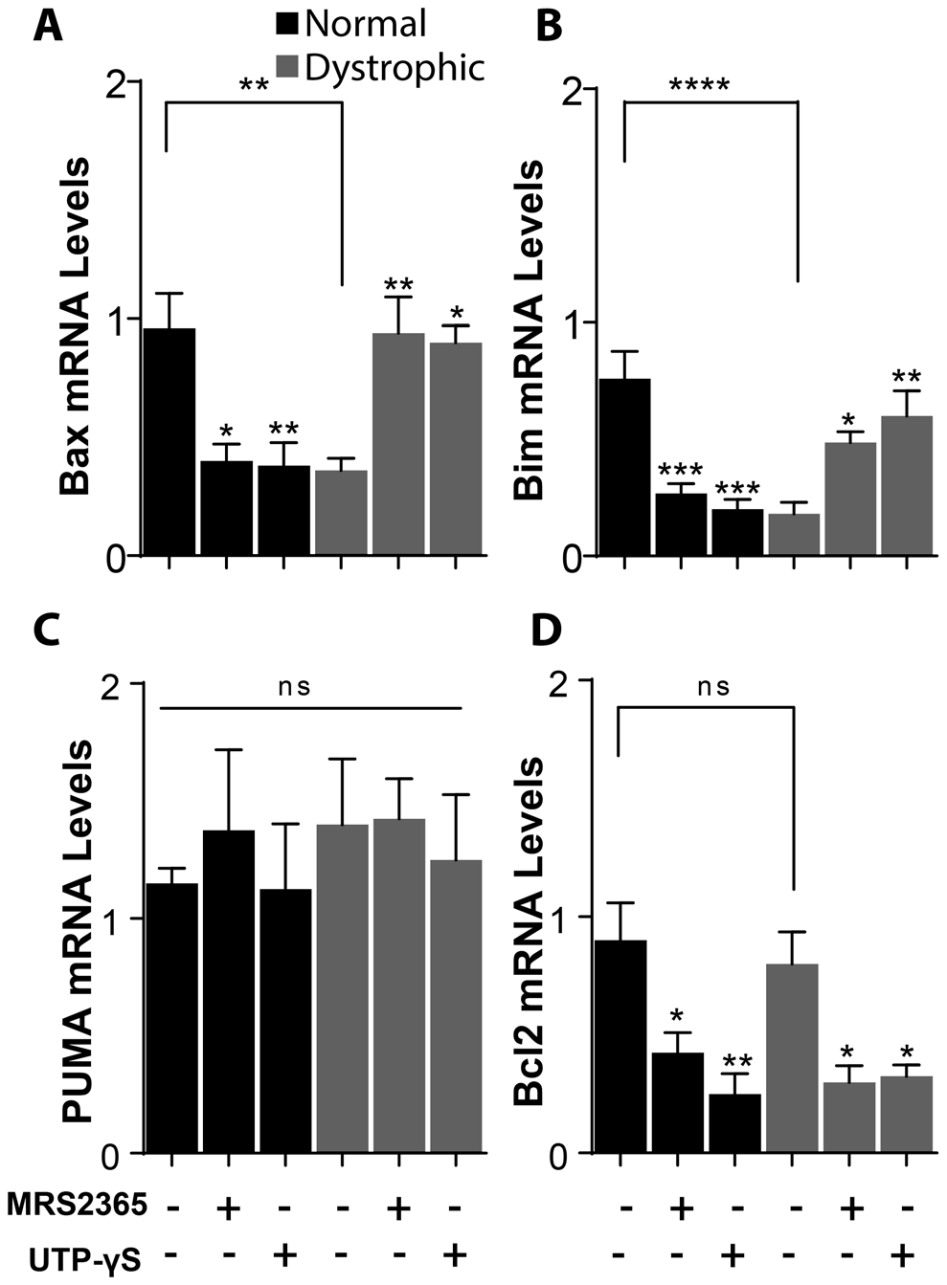
P2Y receptors activate the expression of genes related with cell death in dystrophic fibers. Normal and dystrophic fibers were stimulated with P2Y1 or P2Y2 agonists for 1(P2Y1 agonist) and 10 µM UTP-γS (P2Y2 agonist) induced a decrease in mRNA levels of Bax (A) and Bim (B) in normal fibers and an increase in the same mRNA levels in dystrophic fibers. mRNA levels of PUMA did not change in any fiber type (C), whereas mRNA levels of Bcl2 were reduced in normal and dystrophic fibers with agonists for both, P2Y1 and P2Y2 (D) (n = 4–6 for each gene). Data were expressed as mean ± SE. *p<0.05,**p<0.01,***p<0.001,****p<0.0001.

To better understand the mechanisms implicated in activation of cell death genes after ATP stimulation in dystrophic muscle fibers we measured the changes in mRNA levels of Bax, Bim, PUMA and Bcl2 after stimulation of fibers with specific agonists of P2Y1 receptor (MRS 2365) and of P2Y2 receptor (UTP-γS). We found that 100 nM of MRS 2365 and 10 µM of UTP-γS produce a decrease in mRNA levels of Bax, Bim and Bcl2 in control muscle fibers and an increase in both Bax and Bim mRNA levels in dystrophic fibers (Fig. 5 A, B and D). No changes were observed in the mRNA levels of PUMA in both fiber types (Fig. 5 C) and Bcl2 mRNA also decreased in dystrophic muscle fibers as in control ones (Fig. 5D). These results suggest that the transcriptional changes observed after ATP stimulation may at least in part occur trough activation of P2Y1 and P2Y2 receptors.

## Discussion

It is known that electrical stimulation has an important role in maintaining skeletal muscle integrity and function (reviewed in [48]–[49]). It can be in some ways homologue to exercise and it has been shown to be determinant in muscle plasticity [50]–[52]. Lack of exercise or denervation lead to rapid and dramatic muscle atrophy and the normal muscular tone appears to have an important trophic function by maintaining normal muscle gene expression [53]–[54]. Apoptosis has been implicated in the development of atrophy in muscle after denervation and disuse [55]–[57] and it has been demonstrated that low frequency electrical simulation can prevent apoptosis in denervated muscles [58]–[59]. Nevertheless a molecular explanation about the molecular mechanisms implicated in this anti-apoptotic effect remains elusive.

We have described that part of the communication between electrical stimulation and muscle gene expression occurs in a protein complex that senses the stimulus (Cav1.1 or DHPR), opens a pathway (Panx1) for ATP release and activates (via P2Y purinergic receptors) a signaling pathway for gene expression [10]–[11]. Our new findings indicate that some genes repressed or activated by this mechanism (Bax, Bim, PUMA and Bcl2) may be important for protecting the muscle fiber against apoptosis, which may take place once the described mechanism is disrupted as occurs in a mouse model for Duchenne muscular dystrophy.

Until now, little attention has been paid to the involvement of signaling pathways other than the known dystrophin associated proteins in the pathogenesis of muscular dystrophy. Nevertheless, in the past years a greater effort has been made to understand, propose and demonstrate new mechanisms involved in the pathogenesis of this disease [60]–[64]. These new efforts are made towards the achievement of new forms of therapy that at least prolong the expectation and quality of life. Several studies have proposed that changes in ATP signaling could be involved in DMD [14], [65] but none of them have implicated a direct function of this pathway in the dystrophic pathology. In this work we show that ATP signaling is altered in dystrophic fibers and that could be involved in the muscle cell loss.

We presented evidence pointing Panx1 as the main route of ATP release in mdx fibers as is the case in normal fibers. However, it is possible that other routes for ATP release in dystrophic fibers exist, because the inhibition of ATP release after mechanical stimulation was higher with a non-selective blocker of hemichannels (oleamide) than with the specific peptide ^10^Panx1 (Fig. 1D). In fact it has been proposed that in damaged or denervated muscle, there is re-expression of connexins that normally are not expressed in adult healthy muscle [66].

We have previously shown that ATP released by adult muscle fibers plays an important role in excitation-transcription coupling, being under the control of Cav1.1 activation [11]. Here, we showed that dystrophic fibers present elevated levels of basal extracellular ATP, while they are unable to activate its release after electrical stimulation as control fibers do. Because of this, dystrophic fibers will be unable to properly activate downstream IP_3_-depending calcium signals and the corresponding transcriptional changes related to this signaling pathway. This “uncoupling” between electrical stimulation and ATP release could be explained by a loss of control over Panx1 channels by Cav1.1, supported by the data showing a reduction in the interaction between Cav1.1 and Panx1 observed in mdx fibers. Possible explanations are a destabilization of the plasma membrane due to absence of dystrophin or the change in the relative amounts of Cav1.1 and Panx1.

Other studies have shown that the absence of dystrophin could alter the correct function of Cav1.1 [67]–[69]. Even if it is not clear whether alteration in Ca^2+^ currents through this L-type Ca^2+^ channel contributes to DMD pathology, the changes observed in the current voltage dependence and in the voltage activated Ca^2+^release (dependent on Cav1.1), clearly suggest that this protein and its related function is altered in mdx muscle fibers. Here we propose that this could also be the case for its function in excitation-transcription coupling.

Downstream effects of electrical stimulation in muscle fibers constitute one of the most important signals for keeping the health and correct function of muscle fibers. Accordingly, a perturbation in signaling pathways relating electrical stimulation with activation of gene transcription in mdx muscle fibers could participate in the development of this disease. Moreover, we present here the first evidence that low frequency electrical stimulation has a protective effect against cell death in control muscle fibers. This effect appears to be mediated by ATP that is released with this type of stimulation, because ATP by itself could induce the same transcriptional changes than 20 Hz electrical stimulation did. Importantly, the uncoupling between electrical stimulation and ATP release in dystrophic fibers deprives these fibers of the beneficial effects of electrical activity. Even if the increased levels of ATP release in mdx fibers induce a basal reduction in pro-apoptotic genes, ATP stimulation induces an increase in the mRNA levels of these genes and, more important, in the levels of activated Bax protein and of cytosolic citochrome c, showing that ATP in fact activates an apoptotic pathway in dystrophic fibers. Dystrophic muscle fibers are indeed subjected to high levels of extracellular ATP, due to the large amounts of ATP released by the surrounding dying cells.

Regardless of the many studies of necrosis in muscular dystrophy [70]–[72], the involvement of apoptosis in the death of muscle fibers in DMD, the presence of apoptosis as an early condition for necrosis has been proposed [24]. It is possible that *in vivo*, high extracellular ATP levels present in dystrophic muscles induce signaling pathways leading the viable fibers to enter in an apoptotic cell death process.

Several P2Y receptors are expressed in adult muscle fibers and even if all of them are susceptible to play a role in activating signaling pathways downstream ATP stimulation, we have seen that P2Y1 and P2Y2 receptors were two of the most expressed ones in adult muscle fibers [73]. The different P2Y (and P2X) receptors have different ATP sensibility, being able to activate different pathways, depending on ATP concentration [74]. In the present work, we show that stimulation with specific agonists to P2Y1 and P2Y2 induce transcriptional changes similar to those elicited by ATP in control and dystrophic fibers. Differences observed for PUMA and Bcl2 between the treatment with the specific agonist and with ATP could relay in the activity of other, yet unstudied P2Y receptors. In the particular case of Bcl2, that did not change with electrical stimulation or ATP and did decrease with agonists for both P2Y1 and P2Y2 in normal and dystrophic fibers, it is possible that a balance between up-regulation and down-regulation of Bcl2 occurs via the activation of different P2Y receptors.

Taken together, the results of the present study represent strong evidence that ATP signaling exerts an important physiological impact on skeletal muscle homeostasis and that disruption of such signaling may lead to important pathological alterations. The absence of functional dystrophin is likely to provoke an alteration in the assembly of the signaling complex, altering the regulation of ATP release. This will interfere with proper gene expression associated with electrical stimuli and will finally induce a loss of muscle function by the induction of apoptosis and muscle fiber death.

By adequately addressing the pathway alteration described here, new approaches to regulate muscle damage in Duchenne muscular dystrophy can be envisaged; for instance, treatment with drugs that will interfere with Cav1.1, Panx1 or P2Y receptors will decrease the effect of increased ATP release and are candidates to normalize the expression of pro-apoptotic genes [75].

## Supporting Information

Figure S1 **Changes in mRNA levels of Bax at different times and doses of ATP.** Normal and dystrophic muscle fibers were stimulated with different concentrations of external ATP and mRNA levels of Bax were measured 1 h after ATP addition (A and C). We observed the maximum decrease in mRNA levels when using a concentration of 100 µM in normal fibers (A), while the maximum values were found for 50 and 100 µM in dystrophic fibers (B) (n = 4). When fibers were stimulated with 100 µM external ATP and mRNA levels of Bax were measured at different times post ATP addition, we observed that the maximum decrease in Bax mRNA levels was observed after 60 min ATP addition (B and D). Data were expressed as mean ± SE,*p<0.05,**p<0.01.
(TIF)
