# Supplementary figures and images for: Electrical Stimuli Are Anti-Apoptotic in Skeletal Muscle via Extracellular ATP. Alteration of This Signal in Mdx Mice Is a Likely Cause of Dystrophy
Source: PLoS One. 2013 Nov 25;8(11):e75340. doi: 10.1371/journal.pone.0075340 (PMC3839923; doi:10.1371/journal.pone.0075340)

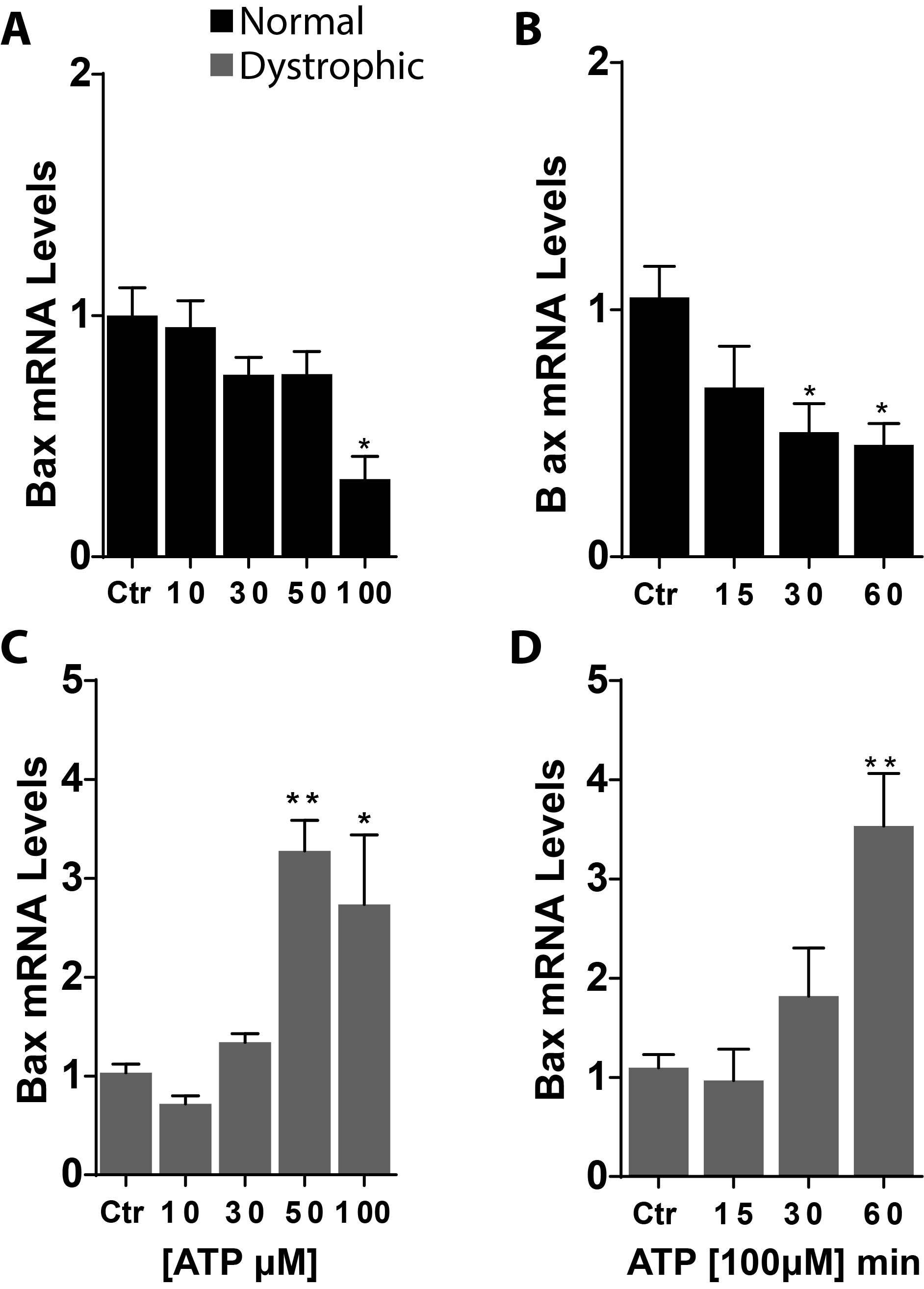

Supplement: Figure S1 — Changes in mRNA levels of Bax at different times and doses of ATP. Normal and dystrophic muscle fibers were stimulated with different concentrations of external ATP and mRNA levels of Bax were measured 1 h after ATP addition (A and C). We observed the maximum decrease in mRNA levels when using a concentration of 100 µM in normal fibers (A), while the maximum values were found for 50 and 100 µM in dystrophic fibers (B) (n = 4). When fibers were stimulated with 100 µM external ATP and mRNA levels of Bax were measured at different times post ATP addition, we observed that the maximum decrease in Bax mRNA levels was observed after 60 min ATP addition (B and D). Data were expressed as mean ± SE,*p<0.05,**p<0.01. (TIF) [file pone.0075340.s001.tif]
